# Nodal Basin Recurrence After Sentinel Lymph Node Biopsy for Melanoma: A Retrospective Multicenter Study in 2653 Patients

**DOI:** 10.1097/MD.0000000000001433

**Published:** 2015-09-11

**Authors:** Lutz Kretschmer, Hans Peter Bertsch, Antonia Zapf, Christina Mitteldorf, Imke Satzger, Kai-Martin Thoms, Bernward Völker, Michael Peter Schön, Ralf Gutzmer, Hans Starz

**Affiliations:** From the Department of Dermatology, Venereology and Allergology, Georg August University of Göttingen, Robert-Koch-Str. 40, D-37075 Göttingen (LK, HPB, KMT, MPS); Department of Medical Statistics, Georg August University of Göttingen, Humboldtallee 32 37073 Göttingen (AZ); Department of Dermatology and Allergy, Hannover Medical School, Carl-Neuberg-Str. 1, 30625 Hannover, Germany (IS, BV, RG); Department of Dermatology, Venereology and Allergology, Klinikum Hildesheim GmbH, Senator-Braun-Allee 33, 31135 Hildesheim, Germany (CM); and Department of Dermatology and Allergology, Klinikum Augsburg, Germany, Sauerbruchstr. 6, D-86179 Augsburg (HS).

## Abstract

The objective of this study was to analyze different types of nodal basin recurrence after sentinel lymph node biopsy (SLNB) for melanoma.

Patients and Methods: Kaplan–Meier estimates and the Cox proportional hazards model were used to study 2653 patients from 3 German melanoma centers retrospectively.

The estimated 5-year negative predictive value of SLNB was 96.4%. The estimated false-negative (FN) rates after 1, 2, 3, 5, and 10 years were 2.5%, 4.6%, 6.4%, 8.7%, and 12.6%, respectively. Independent factors associated with false negativity were older age, fewer SLNs excised, and head or neck location of the primary tumor. Compared with SLN-positive patients, the FNs had a significantly lower survival. In SLN-positive patients undergoing completion lymphadenectomy (CLND), the 5-year nodal basin recurrence rate was 18.3%. The recurrence rates for axilla, groin, and neck were 17.2%, 15.5%, and 44.1%, respectively. Significant factors predicting local relapse after CLND were older age, head, or neck location of the primary tumor, ulceration, deeper penetration of the metastasis into the SLN, tumor-positive CLND, and >2 lymph node metastases. All kinds of nodal relapse were associated with a higher prevalence of in-transit metastases.

The FN rate after SLNB steadily increases over the observation period and should, therefore, be estimated by the Kaplan–Meier method. False-negativity is associated with fewer SLNs excised. The beneficial effect of CLND on nodal basin disease control varies considerably across different risk groups. This should be kept in mind about SLN-positive patients when individual decisions on prophylactic CLND are taken.

## INTRODUCTION

Lymph node excision in melanoma pursues 3 goals: staging, regional disease control, and cure. Before the introduction of sentinel lymph node (SLN) biopsy (SLNB), a so-called delayed lymph node dissection was the clinical standard in many European countries. We performed this type of therapeutic lymphadenectomy on patients with initially unsuspicious lymph nodes who developed clinically enlarged lymph node metastases in the later course of their disease. The advantage of this “wait and watch” strategy was that only those patients with lymphatic metastasis were exposed to the risk of significant morbidity after radical lymphadenectomy.^1^ However, nodal basin recurrence was a frequent complication at the time.^2–6^ In one study, lymph nodes larger than 6 cm led to a failure rate of 80%, compared to 42% for nodes 3 to 6 cm and 24% for nodes smaller than 3 cm.^7^ Cutaneous ulceration, lymph edema, disfigurement, or pains are typical symptoms of nodal basin recurrence that may considerably impair the quality of life of the affected individuals.

Nowadays, owing to the SLN procedure, lymphatic melanoma metastases can be excised early when the nodal tumor burden is low. Two types of nodal basin recurrence are of interest after SLNB: (1) local recurrence after tumor-negative SLNB, which concerns the false-negative (FN) rate and the sensitivity of SLNB as a staging procedure and (2) local recurrence after positive SLNB plus subsequent completion lymph node dissection (CLND), which determines the chances of local disease control in patients with nodal metastases. Improved nodal basin control rates after positive SLNB + CLND might be an important argument in favor of the SLN procedure. Indeed, in a meta-analysis, only 7.5% of the patients undergoing SLNB + CLND experienced recurrences in the same nodal basin.^8^ Unfortunately, however, most of the studies included were limited by their exclusive analysis of percentages of recurrence. In the present retrospective study, we consequently applied the Kaplan–Meier method. This allowed us to provide the cumulative proportion of false-negativity after SLNB for the first time. This approach was predicted to improve the comparability with other studies and to allow for further assessment of risk by using the appropriate statistical methods, such as log-rank tests and the Cox proportional hazards model.

## PATIENTS AND METHODS

### Patients and Clinical Procedures

From 1998 to 2010, a total of 2653 melanoma patients received a successful SLNB at the tertiary-care dermatological clinics of Augsburg, Göttingen, and Hannover, Germany. Clinical and histological data of the patients were collected, using electronic databases. Concerning lymphatic mapping and SLNB, details of our technical approaches and histological methods used have been published elsewhere.^9,10^ In accordance with the recent American Joint Committee on Cancer classification, in this paper the term “SLN metastasis” refers to the presence of melanoma cells in an SLN irrespective of their number and distribution.^11^ We routinely registered the maximum distance of intranodal melanoma cells from the interior margin of the nodal capsule (tumor penetrative depth (TPD)). The underlying S-classification categorizes the TPD into ≤0.30 mm, 0.31 to 1.00 mm, and > 1.00 mm (s1–s3).^9^ Patients with pathologically positive SLNs were offered a CLND, which was carried out according to established standard techniques. In patients with neck metastases, either modified radical neck dissection or different types of selective neck dissection were performed. Axillary dissection comprised dissection of levels I to III. In patients with groin metastases, either an inguinal dissection or the more extended ilioinguinal dissection was performed.

Lymph node recurrence in the same basin after initially negative SLNB was counted as FN result. We recorded both isolated nodal recurrences and nodal recurrences occurring after other kinds of recurrence. Local recurrence after tumor-positive SLNB and subsequent CLND (SLNB + CLND) was defined as any nodal or nonnodal recurrence within the surgical bed of the nodal dissection. The patients were routinely monitored according to national guidelines.^12^ The institutional review board in Göttingen approved this retrospective study.

### Statistics

We recorded the following variables related to patient demographics and tumor parameters: age at the time of primary diagnosis, gender, location of the primary melanoma, Breslow thickness, ulceration of the primary melanoma, pathologic status of the SLN, the TPD into the SLN, the number of tumor-involved lymph nodes, and the pathologic status of the CLND. The outcome variables for which data were collected were melanoma-specific overall survival, time to recurrence in the regional nodal basin, and time to in-transit metastases. The negative predictive value of the sentinel node biopsy and the FN rate were calculated according to their mathematical definitions. Moreover, we provided an “estimated negative predictive value” and an “estimated FN rate”, as calculated by the Kaplan–Meier method. The estimated FN rate was calculated as follows: we created the outcome variable including the time to nodal basin recurrence for the FNs (uncensored observations) and the overall survival time for the SLN-positives (censored observations, event “FN” did not occur). This way, the cumulative proportion of patients with lymph node metastasis diagnosed by SLNB can be estimated using the Kaplan–Meier approach. The “estimated FN rate” was obtained by subtracting this proportion from 100%. Nodal basin recurrence rates among different risk groups were analyzed using the log-rank test and Cox proportional hazards regression models. Individual model covariates were characterized by the adjusted relative risk (RR) and by 95% confidence intervals (95% CI) on the hazard ratio scale. For analysis of metric data comparing two independent groups, the *t* test (for normally distributed variables) or the nonparametric Mann–Whitney test were used. We performed the Mann–Whitney test for ordinal variables. The Kruskal–Wallis Test was used to compare ≥3 independent groups of sampled data. The significance level was set to α = 5% (2-sided). Survival analyses and descriptive statistics were calculated using the software “Statistica” (version 10.0, StatSoft).

## RESULTS

### Risk Profile of the Studied Population

Of the 2653 patients included, 289 (11%) had a primary melanoma on the head or neck, 1021 patients (38%) had truncal melanomas, and 1343 patients (51%) had extremity-located primary tumors. There was a relatively equal distribution of the sexes (51.5% male). The median age was 60.8 years (range 6–93 years); the median Breslow thickness was 1.6 mm (range 0.3 mm–20 mm, mean 2.32 ± 2.1 mm). Of the 2048 patients with available information on ulceration, 25.9% were ulcerated. After the SLN procedure, 691 patients (26%) were assessed as SLN-positive. Of these, 458 (66%) received a CLND. Of the CLNDs, 116 (25.3%) were tumor-positive. In patients undergoing SLNB + CLND, the mean number of excised lymph node metastases was 1.9 ± 1.6. The estimated 5-year survival rates for SLN-negative and SLN-positive patients were 91.8% and 73.8%, respectively (*P* < 0.001).

### Estimated Negative Predictive Value

**FIGURE 1:**
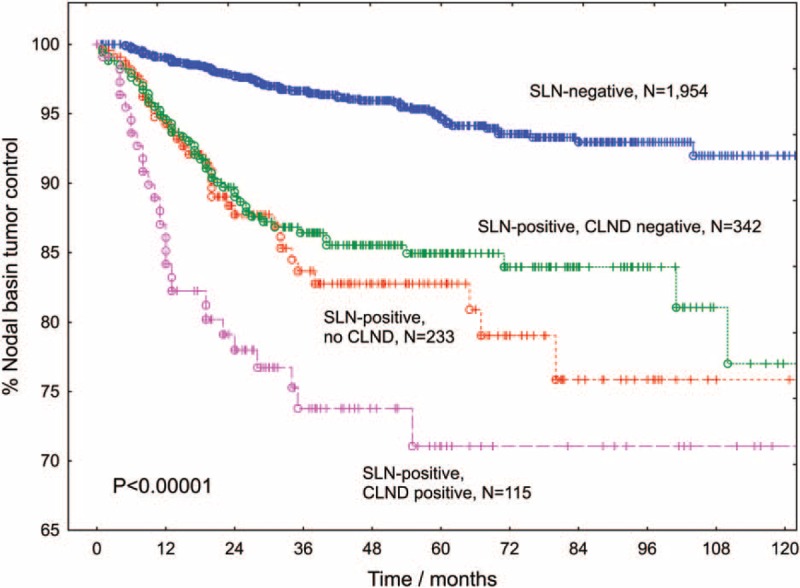
Local control rates after SLN biopsy according to the type of lymph node surgery performed. The blue curve (including all 77 nodal recurrences in the same nodal basin after initially negative SLNB) depicts the “estimated negative predictive value of SLNB” >10 years. The green and the magenta curves demonstrate the probability of a tumor-free nodal basin in the SLN-positive subpopulations with pathologically negative or positive CLND, respectively. The SLN-positive patient group without CLND (red curve) is clearly biased, because it includes, on the one hand, patients who refused radical surgery, elderly patients or patients with increased general morbidity and, on the other hand, patients showing a relatively low nodal tumor burden. CLND = completion lymph node dissections, SLN = sentinel lymph node, SLNB = sentinel lymph node biopsy.

Clinical and histological characteristics of the study population are shown in Table 1, according to the SLN Status. After SLNB, 1965 patients were declared SLN-negative. After a mean follow-up of 52.5 ± 34.8 months, 77 patients had developed clinically evident node metastases in a nodal basin initially determined as SLN-negative. Of these, 49 displayed the enlarged lymph node metastasis as first recurrence. Considering all 77 nodal recurrences, the negative predictive value was 95.9% and the estimated 5-year local control rate was 94.6% (Figure 1, blue curve). Upon exclusion of the 28 patients who developed nodal recurrence after other types of melanoma recurrence, the negative predictive value was 97.7% and the “estimated 5-year negative predictive value” as determined by the Kaplan–Meier method was 96.4%.

**TABLE 1:**
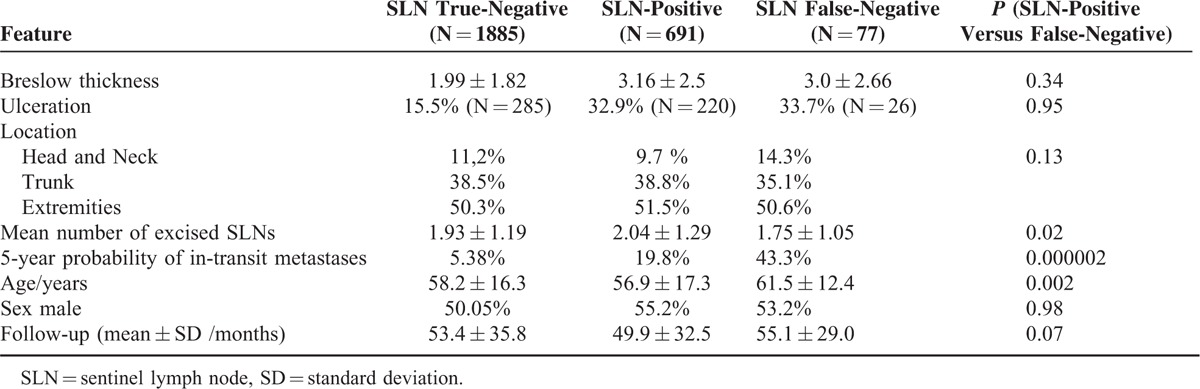
Clinical and Histological Characteristics of the Study Population, According to the SLN Status

### Probability of Diagnosis of Nodal Metastasis by SLNB and Estimated False-Negative Rate

**FIGURE 2:**
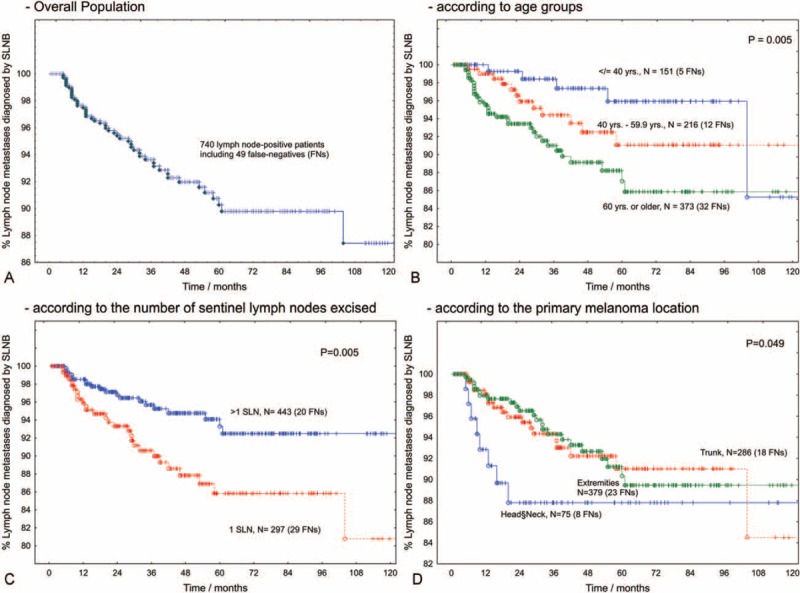
Proportion of node-positive patients diagnosed by sentinel lymph node biopsy (true-positives). During follow-up, the sensitivity of SLNB steadily declines due to the occurrence of nodal recurrences in patients with an initially negative SLNB (FNs, A). Older age (B), the excision of only 1 SLN (C) and head or neck location of the primary melanoma (D) are associated with a lower sensitivity of SLNB and a higher FN rate, respectively. FNs = false-negative results, SLN = sentinel lymph node, SLNB = sentinel lymph node biopsy.

At the time of this analysis, a total of 768 patients had lymph node metastases in a nodal basin explored by SLNB (691 true-positives and 77 FNs including 49 FNs displaying the nodal metastasis as the first recurrence). According to the mathematical definition, the FN rate was 10%, if all nodal recurrences were considered, and 6.4% if only nodal recurrences appearing as the first recurrence were taken into account. Using Kaplan–Meier estimates, the estimated FN rates after 1, 2, 3, 5, and 10 years were 3.6%, 6.5%, 8.9%, 13.9%, and 17.5%, respectively. If only the isolated nodal recurrences were considered, these figures decreased to 2.5%, 4.6%, 6.4%, 8.7%, and 12.6%, respectively (Figure 2A).

In univariate Cox regression analysis, the probability of being FN increased with age (*P* = 0.005) and with a lower number of SLNs excised (*P* = 0.004). The estimated 5-year FN rate was 14% in patients with only 1 SLN, compared to 6.4% in patients with ≥2 SLNs excised. Older age and head or neck location were also associated with a higher FN rate (Figure 2). The estimated FN rate did not significantly depend on the partaking institution, the Breslow index, ulceration, or sex.

In a multifactorial Cox model, we included those factors that reached significance level in univariate analysis. Although the patients’ age attained borderline significance (adjusted RR 1.02 / year (95% CI: 0.998–1.034; *P* = 0.07)), head or neck location of the primary tumor (adjusted RR 2.2 (95% CI: 1.004–4.799; *P* = 0.48)) and a decreasing number of excised SLNs (adjusted RR 0.60 per SLN (95% CI: 0.417–0.874; *P* = 0.007)) turned out to be independent factors predicting false-negative results of SLNB.

Importantly, the FN cases that appeared as a first recurrence displayed a significantly lower 5-year overall survival rate than the SLN-positive patients (46.9% versus 73.5%, *P* < 0.001). As shown in Table 1, the FNs displayed a significantly increased probability of developing in-transit metastases during the course of the disease.

### Probability of Nodal Basin Recurrence After Initially Tumor-Positive SLNB

The 5-year nodal basin recurrence rates for SLN-positive patients without CLND, with tumor-negative CLND, and with tumor-positive CLND were 15.8%, 14.5%, and 28.5%, respectively (Figure 1). One additional observation worthy of note was that patients who developed in-transit recurrences had a nodal basin failure rate of 52%, compared with a local failure rate after CLND of only 10.6% (*P* < 0.001) in patients who did not develop in-transit metastases during follow-up. In turn, the overall probability of in-transit metastases was 58.8% for patients with local recurrences after CLND and 13.9% for patients who did not experience a local recurrence (*P* < 0.001).

### Nodal Recurrence After SLNB + CLND

**FIGURE 3:**
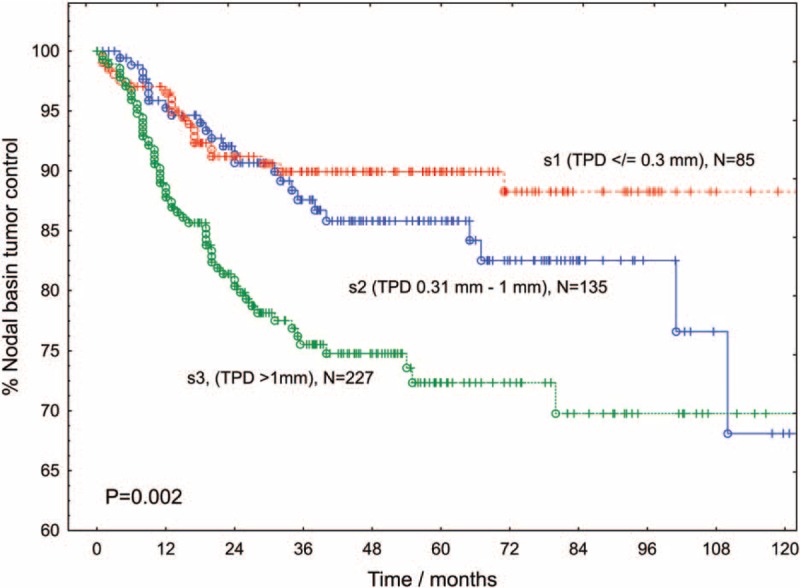
Proportion of definitive local tumor control in the bed of completion lymph node dissection according to TPD into the sentinel lymph node. With respect to the s-category, the proportions of tumor-positive CLNDs for patients with SLNs categorized as s1, s2, or s3 were 9.3%, 11.0% and 39.0%, respectively (*P* < 0.001). CLND = completion lymph node dissections, TPD = tumor penetration depth.

Overall, the 5-year local recurrence rate after SLNB + CLND was 18.5%. We estimated 5-year local-failure rates for axilla, groin, and neck as 17.2%, 15.5%, and 44.1%, respectively. The higher nodal basin recurrence rate after neck-dissection was significant (*P* < 0.001). The probability of nodal basin recurrence increased with Breslow thickness (*P* = 0.007) and with age (*P* = 0.009). The nodal basin recurrence rate was also increased in patients with ulcerated primary tumors (*P* < 0.001). With respect to the SLN-related factors, deeper penetration of the metastasis into the SLN predicted the recurrence after positive SLNB + CLND (*P* = 0.006, Table 2, Figure 3). Tumor involvement of >2 SLNs (recurrence probability 32.2%, *P* = 0.009) and pathologically positive CLND (recurrence probability 28.5%, *P* = 0.005, Figure 2) also predicted local failure after positive SLNB + CLND. Clinical institution and gender were nonsignificant. Local recurrence rates after SLNB + CLND and the respective positivity rates of CLND for clinically relevant subgroups are displayed in Table 1.

**TABLE 2:**
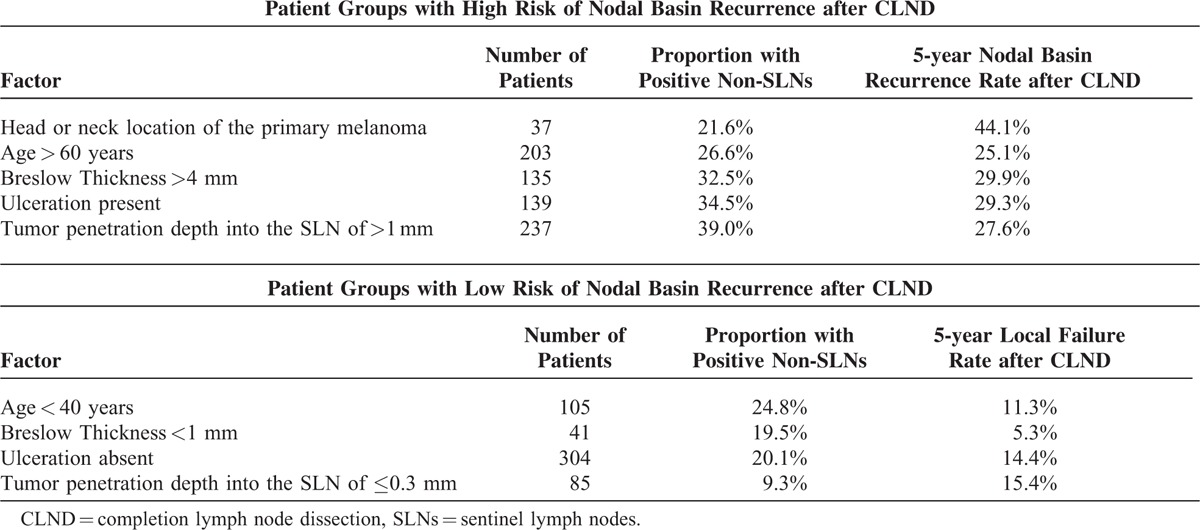
Patient Groups According to Their Probabilities of Pathologically Positive Non-SLNs and of Local Recurrence after Completion Lymph Node Dissection

Using multivariate analysis, we showed that age, ulceration, head or neck location of the primary tumor, and the tumor penetration depth into the SLN were significant independent risk factors of nodal basin recurrence (Table 3). Instead of the tumor penetration depth, we successively included 2 other nodal risk factors in the Cox model. In these models, metastatic involvement of >2 SLNs (adjusted RR 1.7 (95% CI: 1.1–2.8; *P* = 0.03)) and a tumor-positive CLND (adjusted RR 1.8 (95%CI: 1.1–2.9; *P* = 0.02)) were also significant predictors of local recurrence following CLND. Clinical institution, gender, and Breslow thickness were nonsignificant in these models.

**TABLE 3:**
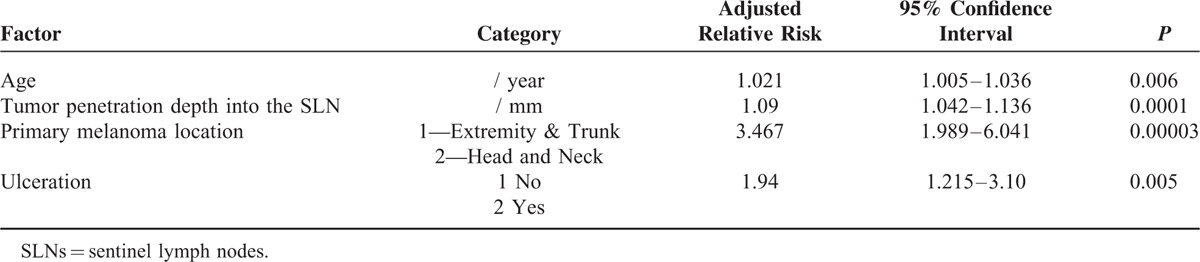
Multivariate Analysis of the Time to Local Recurrence after Completion Lymph Node Dissection (437 Cases with Complete Data, Stepwise Variable Selection)

## DISCUSSION

In their fundamental work, Morton et al^13^ established the basis for SLNB in melanoma. Using confirmative, immediate CLND as a gold standard, they found a negative predictive value of 99% and an FN rate of 4.7%. This led to the abandonment of confirmative CLND in SLN-negative patients. Since then, many authors have identified the FN cases through observation of recurrences after negative SLNB and high negative predictive values between 94.2% and 98.5% have been reported.^8^ Using the Kaplan–Meier method, Nowecki et al^14^ determined an estimated 5-year “nodal basin control rate” of 91.5%. We observed a 5-year nodal basin control rate of 96.4%, supporting the high negative predictive value of the SLN procedure after long-term follow-up. Related to the initially SLN-negative subpopulation, the probability of nodal recurrence depended on Breslow thickness and ulceration of the primary tumor, that is, on the same risk factors that predict SLN metastasis. Jones et al^15^ demonstrated that the male sex is an additional risk factor of nodal recurrence after initially negative SLNB.

As counterpart to the sensitivity of SLNB, the FN rate is defined as the proportion of node-positive patients who had a tumor-negative SLNB.^16^ The reported FN rates vary widely, namely between 0% and 34%.^8^ Obviously, defining the FN rate as a simple fraction makes it difficult to compare the results from different studies. To improve upon this, we introduce the statistically preferable Kaplan–Meier method for estimating the cumulative proportion of node-positive patients diagnosed by SLNB. This allows for an estimation of the false-negativity over time, even when patients drop out or are studied for different lengths of time. A large meta-analysis demonstrated higher FN rates in studies with a higher average quality score.^8^ Our study delivers a possible explanation for this paradoxical finding showing that the FN rate steadily increases over time. Some authors have claimed decreasing FN rates with greater experience.^17,18^ Such statements have to be handled with caution because the patients treated first have been followed for the longest period of time. This alone increases the probability of being FN. FN results after SLNB have been explained by technical factors and by the biology of the disease.^19^ Errors in lymphoscintigraphy, surgery, or pathology have been demonstrated and intensely discussed.^20–24^ This is the first study showing that the FNs have a lower number of SLNs excised when correctly compared with the SLN-positive patients. Using hybrid single-photon emission computed tomography-computed tomography (SPECT-CT), Stoffels et al^25^ demonstrated an increase of the number of excised SLNs. This might be especially useful to improve the FN rates in the head and neck area. The association of false-negativity with age, head, or neck location and with in-transit metastases has already been described by means of univariate analyses.^23,26–28^ Using multivariate analysis, we identified 3 independent predictors of the FN rate: the number of SLNs, head, or neck location of the primary melanoma, and older age.

We observed a relatively low 5-year FN rate of 8.6%, which corresponds with an SLN-positivity rate of 26%. In a new study from the National Cancer Data Base including 33,639 SLNB patients, surgery at hospitals with lower-than-expected SLNB positivity rates was associated with decreased survival.^29^ The impact of false-negativity on overall survival remains controversial. In our study, the FNs did not significantly differ from the SLN-positive patients with respect to Breslow thickness, ulceration, or gender. In agreement with one previous study,^20^ but in contrast to other studies,^14,23,30^ we observed a significantly lower overall survival of the FN patients, compared with SLN-positive patients.

Studies comparing SLNB + CLND with delayed lymph node excision have demonstrated a survival benefit of early over delayed lymph node excision in the subgroup of patients with nodal metastases.^31^ However, a significant contribution to the improved outcome resulting from the prophylactic CLND has not been proven yet. Therefore, the capacity of a CLND to provide definitive tumor control in the bed of a lymph node dissection is of prime importance. In a meta-analysis of studies providing percentages of recurrences, only 7.5% of the patients developed nodal recurrences after positive SLNB + CLND. Using Kaplan–Meier estimates, we obtained a 5-year nodal basin recurrence rate of 18.5%. Studies dealing with therapeutic lymphadenectomy dissection of clinically enlarged lymph node metastases demonstrated nodal basin recurrence rates varying between 25% and 52%.^2–7,32^ Thus, the nodal basin recurrence probabilities after SLNB + CLND seem to be better than those reported after therapeutic lymphadenectomy for enlarged node metastases. However, even after SLNB + CLND, some subgroups of patients displayed a high probability of nodal basin recurrence (Table 2). In the actual target group of CLND, that is, patients with positive non-SLNs, the goal of definitive local disease control was not achieved in 28.5% of cases. The number of lymph node metastases was positively associated with nodal basin relapse. Using multivariate analysis, we estimated a 3.5-fold increased relative risk of nodal recurrence for patients with neck dissections (recurrence rate 44%). Guggenheim et al^33^ observed neck recurrences in 33% of cases. In view of the diversity of the lymphoscintigraphic results in the neck region, the appropriateness of the commonly performed types of neck dissections requires further research. In multivariate analysis, older age, ulceration, and increasing tumor penetration depth into the SLN turned out as additional risk factors of local recurrence after SLNB + CLND (Table 3). In this context, the tumor penetration depth is an outstanding feature, because it is the only SLN-related factor that allows predicting the risk of non-SLN metastases as well as the nodal basin recurrence probability already before a CLND. Importantly, young patients and patients with thin primary tumors had a relatively favorable ratio of tumor-positive CLND to nodal basin recurrence after CLND.

With 768 node-positive patients analyzed statistically, we present one of the largest studies dealing with nodal basin recurrences after SLNB. Some limitations have to be considered: the study has a retrospective design. The extent of neck and groin dissection depended on the personal experience of the treating surgeons and we were not able to differentiate between different types of CLND in these regions. In-transit metastasis as a possible cause of nodal basin recurrence must be questioned, because any melanoma recurrence probably increases the risk of other types of recurrence.

## CONCLUSIONS

We show that false-negativity after SLNB increases over time, indicating that this parameter should be estimated by the Kaplan–Meier method. It is well known that the risk of any nodal basin recurrence depends on the biology of a given tumor and on technical or individual factors. This study adds the observation that the number of SLNs excised is inversely associated with the FN rate. The beneficial effect of CLND on nodal basin disease control varies considerably across different risk groups. This should be kept in mind when individual decisions for or against CLND are taken. From a historical perspective, it appears that the long-term local control rates are superior to the respective results that have been reported after therapeutic lymph node dissection for clinically enlarged lymph node metastases. This assumption is supported by our data showing a significant association between nodal tumor burden and nodal basin recurrence after positive SLNB + CLND.

## References

[1] Kretschmer L Thoms KM Peeters S Postoperative morbidity of lymph node excision for cutaneous melanoma-sentinel lymphonodectomy versus complete regional lymph node dissection. *Melanoma Res* 2008; 18:16–21. 18227703 10.1097/CMR.0b013e3282f2017d

[2] Kissin MW Simpson DA Easton D Prognostic factors related to survival and groin recurrence following therapeutic lymph node dissection for lower limb malignant melanoma. *Br J Surg* 1987; 74:1023–1026. 3690228 10.1002/bjs.1800741122

[3] Shaw JH Rumball EM Complications and local recurrence following lymphadenectomy. *Br J Surg* 1990; 77:760–764. 2383751 10.1002/bjs.1800770715

[4] Monsour PD Sause WT Avent JM Local control following therapeutic nodal dissection for melanoma. *J Surg Oncol* 1993; 54:18–22. 8377499 10.1002/jso.2930540107

[5] Kretschmer L Neumann C Preusser KP Superficial inguinal and radical ilioinguinal lymph node dissection in patients with palpable melanoma metastases to the groin—an analysis of survival and local recurrence. *Acta Oncologica* 2001; 40:72–78. 11321665 10.1080/028418601750071091

[6] Kretschmer L Preusser KP Standardized axillary lymphadenectomy improves local control but not survival in patients with palpable lymph node metastases of cutaneous malignant melanoma. *Langenbeck's Arch Surg/Deutsche Gesellschaft fur Chirurgie* 2001; 386:418–425. 10.1007/s004230100248 11735014

[7] Lee RJ Gibbs JF Proulx GM Nodal basin recurrence following lymph node dissection for melanoma: implications for adjuvant radiotherapy. *Int J Radiat Oncol, Biol, Phys* 2000; 15: 46:467–474. 10661355 10.1016/s0360-3016(99)00431-9

[8] Valsecchi ME Silbermins D de Rosa N Lymphatic mapping and sentinel lymph node biopsy in patients with melanoma: a meta-analysis. *J Clin Oncol* 2011; 29:1479–1487. 21383281 10.1200/JCO.2010.33.1884

[9] Starz H Siedlecki K Balda BR Sentinel lymphonodectomy and s-classification: a successful strategy for better prediction and improvement of outcome of melanoma. *Ann Surg Oncol* 2004; 11 (3 Suppl):162S–168S. 15023745 10.1007/BF02523622

[10] Mitteldorf C Bertsch HP Zapf A Cutting a sentinel lymph node into slices is the optimal first step for examination of sentinel lymph nodes in melanoma patients. *Modern pathology: an official journal of the United States and Canadian Academy of Pathology, Inc* 2009; 22:1622–1627. 10.1038/modpathol.2009.137 19801968

[11] Balch CM Gershenwald JE Soong SJ Final version of 2009 AJCC melanoma staging and classification. *J Clin Oncol* 2009; 27:6199–6206. 19917835 10.1200/JCO.2009.23.4799 PMC2793035

[12] Pflugfelder A Kochs C Blum A S3-guideline “diagnosis, therapy and follow-up of melanoma”—short version. *J German Soc Dermatol* 2013; 11:563–602. 10.1111/ddg.12044 23721604

[13] Morton DL Wen DR Wong JH Technical details of intraoperative lymphatic mapping for early stage melanoma. *Arch Surg* 1992; 127:392–399. 1558490 10.1001/archsurg.1992.01420040034005

[14] Nowecki ZI Rutkowski P Nasierowska-Guttmejer A Survival analysis and clinicopathological factors associated with false-negative sentinel lymph node biopsy findings in patients with cutaneous melanoma. *Ann Surg Oncol* 2006; 13:1655–1663. 17016755 10.1245/s10434-006-9066-0

[15] Jones EL Jones TS Pearlman NW Long-term follow-up and survival of patients following a recurrence of melanoma after a negative sentinel lymph node biopsy result. *JAMA Surg* 2013; 1–6. 10.1001/jamasurg.2013.1335 PMC3856253 23325294

[16] Nieweg OE Tanis PJ de Vries JD Sensitivity of sentinel node biopsy in melanoma. *J Surg Oncol* 2001; 78:223–224. 11745813 10.1002/jso.1156

[17] Veenstra HJ Wouters MW Kroon BB Less false-negative sentinel node procedures in melanoma patients with experience and proper collaboration. *J Surg Oncol* 2011; 104:454–457. 21538361 10.1002/jso.21967

[18] van der Ploeg AP Haydu LE Spillane AJ Outcome following sentinel node biopsy plus wide local excision versus wide local excision only for primary cutaneous melanoma: analysis of 5840 patients treated at a single institution. *Ann Surg* 2014; 260:149–157. 24633018 10.1097/SLA.0000000000000500

[19] Sondak VK Nonsentinel node metastases in melanoma: do they reflect the biology of the tumor, the lymph node or the surgeon?: Editorial to Accompany Ghaferi et al., ASO-2009-03-0312.R1. *Ann Surg Oncol* 2009; 16:2965–2967. 19669838 10.1245/s10434-009-0667-2 PMC2766452

[20] Testori A De Salvo GL Montesco MC Clinical considerations on sentinel node biopsy in melanoma from an Italian multicentric study on 1,313 patients (SOLISM-IMI). *Ann Surg Oncol* 2009; 16:2018–2027. 19132446 10.1245/s10434-008-0273-8

[21] Nieweg OE Veenstra HJ False-negative sentinel node biopsy in melanoma. *J Surg Oncol* 2011; 104:709–710. 21792944 10.1002/jso.22043

[22] Kretschmer L Peeters S Beckmann I Intraoperative detection of sentinel lymph nodes in cutaneous malignant melanoma—blue dye alone versus blue dye plus gamma detection. *J German Soc Dermatol* 2005; 3:615–622. 10.1111/j.1610-0387.2005.05735.x 16033480

[23] Scoggins CR Martin RC Ross MI Factors associated with false-negative sentinel lymph node biopsy in melanoma patients. *Ann Surg Oncol* 2010; 17:709–717. 19967459 10.1245/s10434-009-0858-x

[24] Manca G Rubello D Romanini A Sentinel lymph node mapping in melanoma: the issue of false-negative findings. *Clin Nucl Med* 2014; 20: 10.1097/RLU.0000000000000366 24561692

[25] Stoffels I Boy C Poppel T Association between sentinel lymph node excision with or without preoperative SPECT/CT and metastatic node detection and disease-free survival in melanoma. *JAMA* 2012; 12: 308:1007–1014. 22968889 10.1001/2012.jama.11030

[26] Carlson GW Page AJ Cohen C Regional recurrence after negative sentinel lymph node biopsy for melanoma. *Ann Surg* 2008; 248:378–386. 18791358 10.1097/SLA.0b013e3181855718

[27] Caraco C Marone U Celentano E Impact of false-negative sentinel lymph node biopsy on survival in patients with cutaneous melanoma. *Ann Surg Oncol* 2007; 14:2662–2667. 17597345 10.1245/s10434-007-9433-5

[28] Miller MW Vetto JT Monroe MM False-negative sentinel lymph node biopsy in head and neck melanoma. *Otolaryngol Head Neck Surg* 2011; 145:606–611. 21659495 10.1177/0194599811411878

[29] Kinnier CV Paruch JL Dahlke AR Adjusted hospital sentinel lymph node positivity rates in melanoma: a novel potential measure of quality. *Ann Surg* 9000; Publish Ahead of Print. 10.1097/SLA.0000000000001052 26488806

[30] McDonald K Page AJ Jordan SW Analysis of regional recurrence after negative sentinel lymph node biopsy for head and neck melanoma. *Head Neck* 2013; 35:667–671. 22847953 10.1002/hed.23013

[31] Morton DL Thompson JF Cochran AJ Final trial report of sentinel-node biopsy versus nodal observation in melanoma. *N Engl J Med* 2014; 13: 370:599–609. 24521106 10.1056/NEJMoa1310460 PMC4058881

[32] Kretschmer L Sahlmann CO Bardzik P Individualized surgery: gamma-probe-guided lymphadenectomy in patients with clinically enlarged lymph node metastases from melanomas. *Ann Surg Oncol* 2013; 20:1714–1721. 23314605 10.1245/s10434-012-2841-1 PMC3618405

[33] Guggenheim MM Hug U Jung FJ Morbidity and recurrence after completion lymph node dissection following sentinel lymph node biopsy in cutaneous malignant melanoma. *Ann Surg* 2008; 247:687–693. 18362633 10.1097/SLA.0b013e318161312a

